# Iron supplementation in children to prevent deficiency and anaemia: A qualitative synthesis

**DOI:** 10.4102/phcfm.v17i1.4825

**Published:** 2025-05-14

**Authors:** Denny Mabetha, Idriss I. Kallon, Marianne Visser, Celeste Naude, Willem Odendaal, Amanda S. Brand, Sara Cooper

**Affiliations:** 1 MRC/Wits Rural Public Health and Health Transitions Research Unit (Agincourt), School of Public Health, Faculty of Health Sciences, University of the Witwatersrand, Johannesburg, South Africa; 2 Health Systems Research Unit, South African Medical Research Council, Cape Town, South Africa; 3 Centre for Evidence-based Health Care, Division of Epidemiology and Biostatistics, Faculty of Medicine and Health Sciences, Stellenbosch University, Cape Town, South Africa; 4 Department of Psychiatry, Faculty of Medicine and Health Sciences, Stellenbosch University, Cape Town, South Africa; 5 Cochrane South Africa, South African Medical Research Council, Cape Town, South Africa; 6 School of Public Health and Family Medicine, University of Cape Town, Cape Town, South Africa; 7 Department of Global Health, Faculty of Medicine and Health Sciences, Stellenbosch University, Cape Town, South Africa

**Keywords:** acceptability, anaemia, children, equity, feasibility, low and middle-income countries, iron-deficiency anaemia, qualitative evidence synthesis

## Abstract

**Background:**

Iron deficiency anaemia in young children is a major problem globally, particularly in low- and middle-income countries (LMICs). The World Health Organization (WHO) recommends preventive oral iron supplements to reduce the prevalence of iron deficiency and anaemia in high-prevalence settings.

**Aim:**

To conduct a qualitative evidence synthesis exploring the factors influencing the acceptability, feasibility and equity of preventive oral iron supplementation in young children for the Global Evidence, Local Adaptation (GELA) project, which supports the development of evidence-informed, locally relevant guideline recommendations in three sub-Saharan countries.

**Method:**

We searched MEDLINE, Epistemonikos, CINAHL and PsycInfo from inception to 07 July 2023 for eligible studies. We synthesised the data using thematic analysis and assessed the methodological quality of the studies (using an adaptation of the Critical Appraisal Skills Programme tool) and confidence in the review findings (using GRADE-CERQual).

**Results:**

We included six studies, five from LMICs. Findings indicated knowledge and perceptions about iron supplementation, as well as relationships with intervention providers, can have a beneficial or detrimental influence on caregiver acceptance (moderate to high confidence); caregiver acceptance may be negatively affected by a lack of reliable information but can potentially be enhanced through community-based education (moderate confidence); healthcare workers’ knowledge, resources and support may improve the feasibility of intervention provision (moderate confidence) and socio-economic challenges around access to the intervention may adversely affect equity (low confidence).

**Conclusion:**

A complex interplay of contextual factors may impact the provision and uptake of preventive oral iron supplementation in young children.

**Contribution:**

This work provides insights into how preventative oral iron supplementation might be contextually tailored.

## Introduction

Anaemia in infants and young children is a major public health problem globally, particularly in low- and middle-income countries (LMICs).^1,2^ In children under 5 years, anaemia prevalence is 56.6% in LMICs;^3,4^ higher than the 42% global prevalence.^3^ In South Africa (SA), the prevalence of anaemia in children aged 6 to 59 months has remained high since the 2000s, with an estimated prevalence ranging between 40% and 52% and 22% and 54% among infants aged 1–13 months and 24–59 months, respectively, in 2021.^4^ Anaemia results from multiple causes (e.g. iron deficiency, infections such as malaria, haemoglobinopathies, malaria and bone marrow disorders);^5,6,7^ however, around 42% of global anaemia cases in children under 5 years are estimated to result from iron deficiency,^7^ with a range of complex factors causing, and in some cases, interacting to cause iron deficiency.^8^

Iron is needed to support physical growth, rapid brain development and early learning capacity,^9,10^ with the first 2 years of a child’s life often a ‘tipping point’ for developing iron deficiency. Meeting the iron requirements of infants and young children is challenging in LMICs such as SA, where dietary diversity is poor and household food insecurity is prevalent.^11^ Complementary infant and young child feeding practices in many areas of SA are often suboptimal, with many infants and young children being unable to achieve their required dietary intakes of key micronutrients, including iron, from the foods they can typically access.^11,12^

Preventive oral iron supplementation is one of the strategies employed to reduce the prevalence of iron deficiency and anaemia in settings where anaemia prevalence is high (≥ 40%). Oral iron supplementation provided to infants and young children aged 6–23 months at a daily dosage of 10 mg to 12.5 mg for three consecutive months in a year has been shown to reduce the risk of anaemia, iron deficiency and iron deficiency anaemia (IDA).^8,13,14^

Against this background and as part of the Global Evidence, Local Adaptation (GELA) project, the question of oral iron supplementation for preventing iron deficiency and anaemia in infants and young children aged 6–23 months in SA was prioritised.^15^ The GELA project aims to produce evidence-informed, contextualised guideline recommendations for newborn and child health in Malawi, Nigeria and South Africa, preferably using best practice methods for guideline adaptation to improve efficiencies in low-resource settings.^16^ Guideline methods within the GELA project were based on Grading of Recommendations Assessment, Development and Evaluation (GRADE)-ADOLOPMENT, an Evidence-to-Decision (EtD) framework-based approach to adopt, adapt create contextualised recommendations from source guidelines and evidence syntheses.^17^ To inform recommendations, the GRADE EtD framework includes assessments of relevant evidence to inform judgements for several criteria, including potential benefits and harms of the intervention, economic impacts, as well as acceptability, feasibility and equity implications of the intervention.^18^

A range of complex factors may affect the acceptability of oral iron supplementation. Similarly, the feasibility and equity of its implementation may be influenced by a complex set of considerations. For example, these supplements can be administered in various forms, for example, syrup or drops,^18^ with varying levels of acceptability.^19^ Furthermore, the acceptability of and adherence to this intervention may be affected by mild gastrointestinal adverse effects.^15,17^

Qualitative research can contribute to a better understanding of these factors as it explores perceptions and practices and how different factors interact to shape these. In evidence-informed decision-making, as facilitated by the GRADE EtD framework, synthesised qualitative evidence should ideally inform considerations around the acceptability, feasibility and equity of interventions.^20,21^

To inform the domains of acceptability, feasibility and equity in the EtD framework and in line with GRADE-ADOLOPMENT, we first conducted a scoping for available qualitative evidence synthesis (QES) that could be used or updated. Our scoping review yielded no appropriate QESes, and consequently, we undertook a new QES that aimed to explore the factors that influence the uptake and provision of oral iron supplementation as a public health intervention in children aged 6–23 months for the prevention of iron deficiency and anaemia.

## Methods

This QES was prospectively registered with the International Prospective Register of Systematic Reviews (PROSPERO) (CRD42023455658). The conduct of the QES was informed by the Cochrane Qualitative Evidence Synthesis: Protocol and Review Template v1.4 and reporting followed the Enhancing Transparency in Reporting the Synthesis of Qualitative Research (ENTREQ) statement.^22,23^ To further refine the scope of this review question, we invited clinicians, implementers and policymakers involved in iron supplementation implementation or work who have an interest in our topic to participate in a structured discussion using the TRANSFER conversation guide.^24^ The TRANSFER approach facilitates collaboration between reviewers and stakeholders from the start of a review process to systematically and transparently consider factors that may influence the transferability of the review findings. These discussions were considered during data analysis and assessing the ‘relevance’ component of our GRADE-Confidence in the Evidence from Reviews of Qualitative.^20^

### Criteria for considering studies for this review

#### Types of studies

We included primary studies that used qualitative study designs, for example, ethnography, phenomenology and qualitative process evaluations. Studies were eligible if they used qualitative methods for data collection (e.g. focussed group discussions, individual interviews and open-ended survey questions) and qualitative methods for data analysis (e.g. thematic analysis, framework analysis and grounded theory). Only peer-reviewed studies were included. Mixed-methods studies were included where it was possible to extract data that were collected and analysed qualitatively. We excluded studies using qualitative methods for data collection but not for analysis. We only included studies in English.

#### Types of participants and study settings

We included studies from any country that included any cadre of healthcare workers (HCWs), healthy young children aged 6–59 months and their caregivers, policymakers, programme managers or any others involved in, or affected by, the intervention. We excluded studies in refugee settings and those that focussed on iron supplementation among sick, malnourished and human immunodeficiency virus (HIV)-infected children; those exclusively reporting perspectives on oral iron supplementation for the treatment of anaemia or iron deficiency and those focussed on the implementation of the intervention within hospital or inpatient settings.

#### Types of interventions

Eligible studies were those that included perspectives on preventive oral iron supplementation in healthy young children. We excluded studies only reporting perspectives on multiple micronutrient powders, lipid-based nutrient supplements and intravenous iron transfusions.

#### Phenomenon of interest

Our phenomena of interest were the views, experiences and practices of participants regarding the uptake and provision of preventive iron supplementation as a public health intervention in young children. Provision and uptake, and their interaction, included but were not limited to, issues pertaining to feasibility, acceptability and equity.

### Search methods for identifying studies

#### Electronic searches

The search strategy was developed by initially drawing on search terms from an effectiveness review conducted in parallel.^25^ The strategy was refined in consultation with an information specialist.

We searched MEDLINE (via PubMed), Epistemonikos, CINAHL (via EBSCOhost) and PsycInfo to identify eligible studies from inception until 07 July 2023. Where applicable, we included a methodological filter for qualitative studies. Online Appendix Table 1-A1 details the MEDLINE search strategy, which we adapted for the other databases.

#### Searching other resources

We hand-searched the reference lists of included studies and key references (i.e. relevant systematic reviews). We screened the references of a recent systematic review reporting effectiveness evidence linked to the intervention to identify any qualitative studies that were associated with the included studies.^25^ We also cross-checked the effectiveness and economic studies that were included in the effectiveness systematic review and economic evaluation carried out as part of the GELA project to identify qualitative studies associated with these studies.

### Study selection

Two reviewers independently screened titles and abstracts of the identified records to evaluate eligibility. Full-text articles of those identified as potentially relevant were retrieved. Two reviewers then assessed these articles independently. Any disagreements were resolved by discussion or, when required, by involving a third reviewer. We included a table listing studies that were excluded at the full-text assessment stage and the main reason for exclusion.

### Data extraction

Firstly, we developed a data extraction form to extract information about the first author, year of publication, programme or intervention, setting, aim and participant details for each included study. Secondly, we extracted all data relevant to the review objective from the ‘Results and Discussion’ sections of primary studies, that is, participants’ views and experiences about the acceptability, feasibility and equity implications of the intervention of interest. One reviewer extracted descriptive and phenomenological data, and another cross-checked this to ensure that all relevant data had been extracted. Disagreements were resolved by discussion or in consultation with a third reviewer.

### Assessing the methodological quality of included studies

We used an adapted version of the Critical Appraisal Skills Programme (CASP) tool to critically appraise the methodological quality of each included study.^26^ One reviewer conducted the assessments for each study, recording the rating and any explanations, which were cross-checked by a second reviewer. Although we planned to resolve disagreements by discussion between the two reviewers or, if required, by involving a third reviewer, it was not necessary because no disagreements occurred. We did not use our quality assessments to exclude studies but instead to support the GRADE-CERQual assessment of our confidence in the review findings.^20^

### Data management and synthesis

Our analysis was carried out using thematic synthesis.^27^ Before coding, all the extracted data were read through for familiarisation. Following an inductive approach, three reviewers coded data, blinded and in duplicate, using Dedoose version 9.0.^28^ Consensus was reached through discussion. Similar initial codes across studies were then grouped into more analytic codes, which were subsequently organised into broader findings with supporting participant quotes through iterative team discussion.

Findings were further categorised into three themes by drawing on three domains of the EtD framework, that is, acceptability, feasibility and equity. Once the findings had been organised into themes, we re-read the included studies to check that all key study findings were captured by the review findings.

### Confidence in review findings

Two reviewers used the GRADE-CERQual approach to assess our confidence in each finding.^29^ GRADE-CERQual assesses confidence in the evidence based on the following four key components: methodological limitations of the studies, coherence of the review findings, adequacy of the data contributing to a review finding and relevance of the included studies to the review question. After assessing each component, we made a judgement about the overall confidence in the evidence supporting the review finding. GRADE-CERQual includes judging confidence as high, moderate, low or very low. As this review was conducted to inform the development of a guideline for iron supplementation in SA, we applied GRADE-CERQual at the country (rather than global) level. That is, we assessed how much confidence we have in each review finding in relation to the SA context. If no studies from SA were found, we would consider whether there was any indirectness and, if so, address this through the ‘relevance’ domain of the GRADE-CERQual.

All review findings that were assessed using GRADE-CERQual were reported in the summary of qualitative findings (SoQF) table,^30^ regardless of their associated level of confidence.

### Differences between qualitative evidence synthesis and prospective registration

As we anticipated very few qualitative primary studies on preventive oral iron supplementation in children aged 6–23 months, we expanded the age range of eligibility to 6–59 months. We excluded a study on refugee children, as we anticipated that perspectives would differ from those of healthy children from non-refugee settings.

### Review author reflexivity

The review team consisted of researchers with diverse backgrounds, including social sciences (S.C., W.O. and I.I.K.), public health (S.C., I.I.K., D.M. and M.V.), health systems (W.O.) and epidemiology (D.M., M.V. and A.B.). All authors were affiliated with health-related departments in universities or public research institutions. While none were directly involved in delivering prophylactic iron supplementation or clinical healthcare, their engagement in this work was informed by prior experience in QES. The team has extensive expertise in evidence synthesis, particularly in QES (D.M., W.O., I.I.K., S.C., M.V., A.B. and C.N.).

Although not clinically involved, several authors (D.M., S.C., C.N. and M.V.) are familiar with prophylactic iron supplementation. Three authors (D.M., C.N. and M.V.) are trained clinical dieticians or nutritionists with expertise in micronutrient supplementation, including iron for anaemia prevention. S.C. has personal experience with taking prophylactic iron supplementation and M.V. has first-hand experience as a caregiver. However, none had conducted research on prophylactic iron supplementation prior to this review, although S.C. had worked on childhood vaccination.

The review authors had various expectations of the review findings. Expected facilitators included HCWs’ knowledge of supplementation benefits, while anticipated barriers included side effects leading to poor adherence and caregiver challenges in administration. The authors also anticipated the review might reveal issues related to acceptability, feasibility and equity, alongside access and supply challenges in healthcare systems.

While the team held no specific opinions on the findings, they aimed to generate evidence that will potentially inform guideline development and raise awareness about anaemia and iron supplementation in SA and other LMICs. Some authors acknowledged that biomedical and public health approaches often oversimplify intervention uptake, treating it as an individual and technical phenomenon. They expressed hope that this review would highlight the social complexities surrounding prophylactic iron supplementation and the needs, values and preferences of caregivers, HCWs and other stakeholders. To ensure reflexivity, during the review process, the authors regularly reflected on their positions, experiences and assumptions, assessing their influence on study selection and data synthesis. These reflections, along with discussions and decisions made during the review, were systematically documented.

## Review findings

### Description of studies

**FIGURE 1:**
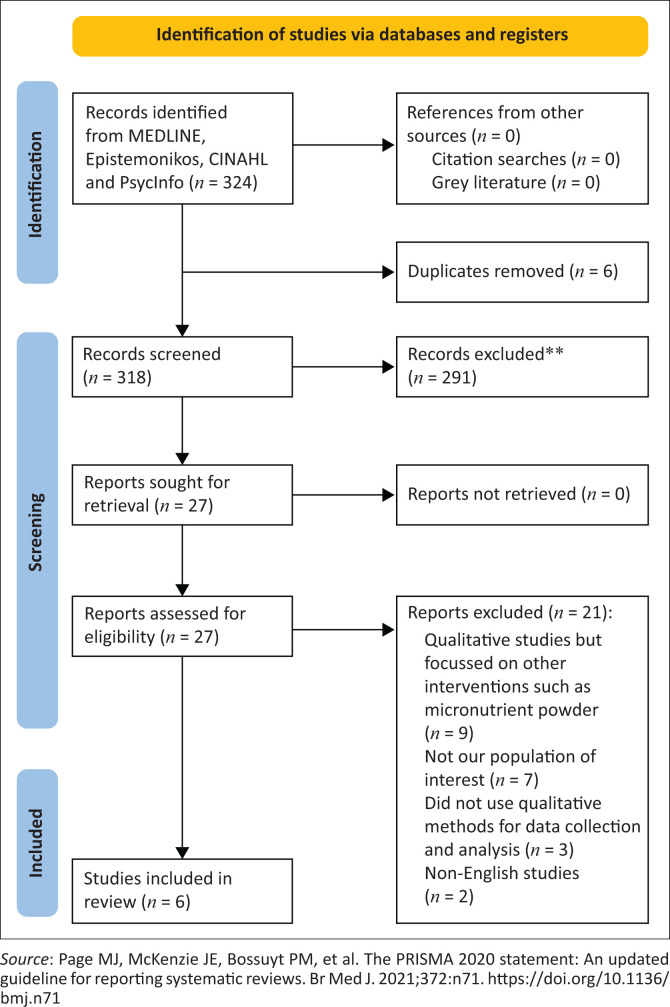
Study flow diagram.

Following deduplication, we screened the titles and abstracts of 318 records. Of these, we assessed 27 full-text articles for eligibility. We excluded 21 full texts with reasons (Table 1) and included six eligible studies in our review (see flow diagram, Figure 1^31^). All studies were published between 2006 and 2021; see Table 2 for a detailed description of the characteristics of the included studies.

**TABLE 1:** List of excluded studies and reasons for exclusion.

Studies	Reasons for excluding
Sammartino (2010)	Language, non-English
Brannon (2017)	Wrong study design
Powers (2020)	Wrong intervention
Bailey-Davis (2018)	Wrong intervention
Endris (2023)	Wrong population
Azeredo (2013)	Language, non-English
Swensen (2001)	Wrong population
Raghu (2021)	Wrong population
Yawson (2017)	Wrong population
Muthuraj (2023)	Wrong population
Wali (2019)	Wrong study design
Jeremias (2023)	Wrong population
Fenta (2020)	Wrong intervention
Ribeli (2022)	Wrong intervention
AzizAli (2021)	Wrong population
Stelle (2021)	Wrong intervention
Berhe (1997)	Wrong intervention
Pantoja-Mendoza (2014)	Wrong intervention
Turk (2017)	Wrong study design
Creed-Kanashiro (2016)	Wrong intervention
Jefferds (2002)	Wrong intervention

**TABLE 2:** Characteristics of included studies.

Study#	References	Study titles	Study aims	Countries, classification by income level, WHO region	Participants	Settings
1	Abu et al.^32^	Qualitative assessments of anaemia-related programs in Ghana reveal gaps and implementation challenges	To conduct multilevel assessments of existing childhood (< 5 years) anaemia prevention and treatment programmes, to elucidate implementation gaps in Ghana using the United Nations International Children’s Emergency Fund (UNICEF)’s conceptual framework of malnutrition as a guide companies, and international agencies	Ghana, lower MIC, Africa	Health service providers, refugee camp management, university faculty, individuals, and organisations involved in food fortification and processing	Universities, local NGOs, government departments, food manufacturing companies and international
2	Kwon et al.^33^	“How Often? How Much? Where From?” Knowledge, Attitudes, and Practices of Mothers and Health Workers to Iron Supplementation Program for Children Under Five in Rural Tamil Nadu, South India	To describe the factors affecting adherence to the iron supplementation programme for children under five in this tribal community of rural Tamil Nadu	India, lower MIC – South-East Asia	Mothers of preschoolers, mothers of children under five, caregivers, VHWs, nurses, teachers, programme officers	NGOs
3	Pierce et al.^37^	Differing perspectives of inner-city parents and paediatric clinicians impact management of iron-deficiency anaemia	To ascertain the viewpoints of parents and paediatric clinicians regarding the identification, understanding and management of anaemia in young children	US, HIC – Americas	Paediatric clinicians, parents (participants and non-participants in the Special Supplemental Nutrition Programme for Women, Infants, and Children)	Paediatric clinics, childcare centres and community agencies, public places such as libraries, parks, training programme, churches and community centres
4	Sguassero et al.^35^	The perspective of primary health care paediatricians regarding childhood anaemia and iron supplementation	To know and analyse the perspective of PHC paediatricians in Argentina regarding the erection of anaemia as a health problem and oral iron supplementation as one of the available interventions for anaemia prevention and management	Argentina, upper MIC – Americas	Paediatricians	Municipal PHC centres
5	Young et al.^34^	Acceptability of multiple micronutrient powders and iron syrup in Bihar, India	To understand LHWs’ perceptions of IFAS and MNPs, ease of delivering them, workload and incentives. Mothers’ perceptions of IFAS and MNPs, reasons for preference of one product over the other and overall preference for the supplements	India, lower MIC – South-East Asia	LHW and mothers	Catchment area of two health centres; Bihar is one of the states in India with the highest burden of undernutrition
6	Yue et al.^36^	Nutritional deficiencies, the absence of information and caregiver shortcomings: A qualitative analysis of infant feeding practices in rural China	To understand current complementary feeding practices in order to identify areas in which these practices are deficient and to identify the factors that impede caregivers from practising proper complementary feeding	China, upper MIC – Western Pacific	Primary caregivers of children	Rural villages; four nationally designated poverty counties in the southern part of Shaanxi Province that have been determined to have high rates of child anaemia

Note: Please see the full reference list of the article, Mabetha D, Kallon II, Visser M, et al. Iron supplementation in children to prevent deficiency and anaemia: A qualitative synthesis. Afr J Prm Health Care Fam Med. 2025;17(1), a4825. https://doi.org/10.4102/phcfm.v17i1.4825, for more information.

HIC, high-income country; IFAS, iron folic acid syrup; LHW, lay health worker; PHC, primary health care; MIC, middle-income country; MNP, multiple micronutrient powders; NGOs, non-governmental organisations; VHW, village health worker; US, United States of America.

#### Study setting

Three studies were conducted in LMICs: one in Ghana^32^ and two in India.^33,34^ Two studies were conducted in upper-middle-income countries (Argentina, China),^35,36^ and one in a high-income country (United States [US]).^37^ Four of the six studies were conducted in settings with a high burden of iron deficiency and anaemia.^34,35,36,37^

#### Participants

Most studies included perspectives of caregivers (mothers and grandparents),^33,34,36,37^ and two of these focussed on the perspectives of caregivers only.^34,36^ Four studies also included the views of HCWs (e.g. nurses and paediatricians) and lay health workers (LHWs).^32,33,35,37^ Of these, one focussed exclusively on the perceptions of paediatricians.^35^ All included studies reported perspectives around preventive iron supplementation in the context of children aged 6 months to 5 years. Some also included perspectives around oral iron supplementation when it was being used for the treatment of anaemia and/or iron deficiency.^27,32,33,34^ Other participants in the studies included programme officers and community stakeholders.^32,33^

#### Methodology

All studies employed in-depth interviews for data collection. Five studies also used focussed group discussions,^33,34,35,36,37^ and one included observational methods for data collection.^32^

### Methodological quality of studies

Most of the studies provided detailed descriptions of the settings, data collection and analysis and supported the findings with evidence; however, several did not adequately describe their sampling strategies and provided no or minimal evidence of researcher reflexivity. Details of the assessments of methodological quality for each included study can be found in Online Appendix Table 2-A1.

### Confidence in review findings

Our confidence in the evidence was assessed based on the four key components of GRADE-CERQual. Based on this approach, we had high confidence in four review findings. We had moderate confidence in six findings and low confidence in one. We did not identify any findings that had limited relevance for the SA context; therefore, GRADE-CERQual judgements are relevant for both the local and global context. Explanations of the GRADE-CERQual assessment for each review finding are shown in the full evidence profile (Online Appendix Table 3-A1).

#### Methodological limitations of studies

Most studies had methodological limitations such as insufficient reporting of setting, sampling strategy and data analysis, as well as a lack of reflexivity and evidence to support claims.

#### Coherence of review findings

We downgraded some findings because of concerns about coherence, in most cases because of some ambiguous data.

#### Adequacy of data contributing to findings

In cases where there was strong evidence for some aspects of the review finding but insufficient data to support other aspects, we downgraded the finding.

#### Relevance of included studies

We did not downgrade any findings because of relevance.

### Findings

We categorised 11 findings into three themes, drawing on the criteria of the EtD framework, namely the factors influencing the acceptability, feasibility and equity of oral iron supplementation in young children (Table 3).

**TABLE 3:** Summary of qualitative findings†.

Summary of review findings	Studies contributing to the review findings	GRADE-CERQual assessment of confidence in the evidence	Explanation of GRADE-CERQual assessment
**Theme 1: Barriers and facilitators to caregivers’ acceptability of iron supplementation as prevention of anaemia and iron deficiency among young children**			
**Finding 1: Caregivers’ limited knowledge of anaemia and iron supplementation** Several caregivers had limited knowledge of anaemia in children, including what it is, its symptoms and its causes, and the role of iron supplementation in its prevention and management. For some caregivers, this limited knowledge reduced their acceptance of iron supplementation for children and may have resulted in them administering lower dosages than recommended	Kwon et al.^33^; Pierce et al.^34^; Yue et al.^36^	High	
**Finding 2: Lack of access to reliab le information on anaemia, nutrition and iron supplementation** Some caregivers raised concerns about their lack of access to reliable information on anaemia and nutrition, reportedly often relying on their own experiences or those of their families and friends or the media as sources of information.	Yue et al.^36^	Moderate	Downgraded because of minor concerns about methodological limitations and serious concerns about adequacy
**Finding 3: Caregivers’ perceived benefits and harms of iron supplements** Some caregivers’ acceptance of iron supplementation was influenced by their perceptions of its benefits and harms. Various caregivers understood the importance of iron supplementation for their children, which may have contributed to enhancing their acceptance of it. Others were concerned about the side effects and complications of iron supplementation, which may have contributed to reducing their acceptance of it or their decision to stop giving it to their children	Kwon et al.^33^; Pierce et al.^37^; Sguassero et al.^35^; Young et al.^34^	High	
**Finding 4: Iron supplementation palatability or tolerability** Some caregivers’ perceptions of the lack of palatability or tolerability of iron supplementation in children reduced their acceptance of it or contributed to them preferring particular types of iron supplementation (e.g. syrups rather than powders)	Kwon et al.^33^; Pierce et al.^37^; Sguassero et al.^35^; Young et al.^34^	High	
**Finding 5: Relationships between HCWs and caregivers** The relationships between HCWs and caregivers may influence caregivers’ acceptance of iron supplementation. Good relationships and trust, whereby HCWs provide caregivers with support and access to iron supplementation, enhanced some caregivers’ acceptance of, or adherence to, iron supplementation. In contrast, poor relationships, whereby caregivers feel they received inadequate support, advice or provision of supplementation, or where there were cultural and linguistic differences, reduced some caregivers’ acceptance of, and adherence to, iron supplementation	Kwon et al.^33^; Pierce et al.^37^; Sguassero et al.^35^; Yue et al.^36^	High	
**Finding 6: Community-based education and support** Various HCWs suggested that community-based education and support, such as workshops, mass education campaigns and peer support groups, could help raise awareness and address concerns about iron supplementation, and in turn, help enhance caregiver acceptance and uptake of iron supplementation	Sguassero et al.^34^	Moderate	Downgraded because of minor concerns about methodological limitations, minor concerns about coherence (ambiguous data) and moderate concerns about adequacy
**Theme 2: Barriers and facilitators to the feasibility of iron supplementation in the prevention of anaemia and iron deficiency among young children**			
**Finding 7: HCWs’ knowledge of anaemia and iron supplementation** Many HCWs had an adequate understanding of anaemia, including its burden, diagnosis, causes and risk factors in children, which reportedly enhanced their ability to manage iron deficiencies in their settings	Abu et al.^32^; Pierce et al.^37^; Sguassero et al.^35^	Moderate	Downgraded because of moderate concerns about methodological limitations, moderate concerns about coherence (ambiguous data), and minor concerns about adequacy
**Finding 8: Lack of adequate resource s and support for HCWs** A lack of resources, support and supervision, including inadequate remuneration, time, supplies and a lack of easily accessible supervisory structures, negatively impacted the motivation and performance of HCWs, particularly LHWs such as VHWs, in iron supplementation delivery	Kwon et al.^33^	Moderate	Downgraded because of minor concerns about methodological limitations, moderate concerns about coherence (ambiguous data) and moderate concerns about adequacy
**Finding 9: Integration of ir on supplementation into routine healthcare** Some HCWs suggested that anaemia prevention and iron supplementation uptake could be enhanced by integrating it into everyday consultations between HCWs and caregivers	Pierce et al.^37^; Sguassero et al.^35^	Moderate	Downgraded because of moderate concerns about methodological limitations, minor concerns about coherence (ambiguous data), and serious concerns about adequacy
**Finding 10: Integration of iron supplementation into LHW programmes and other community-based activities** Local stakeholders, including some HCWs, teachers and a programme officer, suggested that anaemia prevention and iron supplementation uptake could be enhanced by integrating it into LHW programmes and other community-based activities	Abu et al.^32^; Kwon et al.^33^	Moderate	Downgraded because of minor concerns about methodological limitations, minor concerns about coherence (ambiguous data) and serious concerns about adequacy
**Theme 3: Equity issues shaping iron supplementation delivery and uptake**			
**Finding 11: Socio-economic challenges influencing supply and access** Caregivers living in resource-limited settings faced several socio-economic challenges in accessing iron supplementation, including lack of money to purchase iron supplements, long distances from health facilities, transport costs and logistics and limited time, which in turn contributed to irregular or limited usage of iron supplementation among many of these caregivers	Abu et al.^32^, Kwon et al.^33^, Sguassero et al.^35^	Low	Downgraded because of moderate concerns about methodological limitations, moderate concerns about coherence (ambiguous data) and moderate concerns about adequacy

Note: Please see the full reference list of the article, Mabetha D, Kallon II, Visser M, et al. Iron supplementation in children to prevent deficiency and anaemia: A qualitative synthesis. Afr J Prm Health Care Fam Med. 2025;17(1), a4825. https://doi.org/10.4102/phcfm.v17i1.4825, for more information.

GRADE CERQual, grading of recommendations assessment, development and evaluation- confidence in evidence from reviews of qualitative research; HCWs, healthcare workers; LHW, lay health worker.

† , GRADE-CERQual assessments around relevance were made in relation to the SA context; however, we did not identify any findings that had limited relevance for the SA context, and therefore, GRADE-CERQual judgements are relevant for both the local and global context.

#### Theme 1: Barriers and facilitators to caregivers’ acceptability of iron supplementation as prevention of anaemia and iron deficiency among young children

**Finding 1: Caregivers’ limited knowledge of anaemia and iron supplementation (high confidence):** Three studies revealed that caregivers had little knowledge and understanding of anaemia, including its causes and symptoms.^32,35,36^ For example, some caregivers in China gave inaccurate responses when asked to define anaemia:

‘[*A*]naemia? … Doesn’t that mean you don’t have enough blood?’ (Mother, China, Yue et al.^36^)

‘I think you have anaemia when you have low blood pressure and are a little dizzy.’ (Mother, China, Yue et al.^36^)

Others had heard of anaemia or could describe it using common terms such as ‘low blood’; however, they had limited or no understanding of its pathology. For example, when asked about the causes of anaemia, a mother in the US indicated that anaemia was caused by bad food choices ‘too much junk food’ (Parent, US, Pierce et al., 2006), while others mentioned that it resulted from heredity ‘runs in the family’ (Parent, US, Pierce et al., 2006). Similarly, although some mothers in the Indian study showed some knowledge of anaemia, HCWs raised concerns regarding most mothers’ limited understanding of anaemia.^33^ In addition to poor knowledge of anaemia, caregivers did not understand the link between anaemia and the nutritional status of their infants, nor the role of micronutrients and nutrition in anaemia prevention.

Two studies,^33,36^ one each conducted in China and India, revealed that caregivers’ poor knowledge of micronutrients, including IDA management, resulted in them rejecting iron supplementation. For example, a study in China found that most caregivers were unwilling to give micronutrients to their children because of their little understanding of the role of micronutrients in their children’s health:

‘We don’t dare give the baby anything to eat that we’re not familiar with. Even if people say it’s good for him.’ (Mother, China, Yue et al.^36^)

‘When the baby was less than one-year-old I gave him some vitamin supplements, but after that I stopped. After one-year-old the kid doesn’t need supplements. As soon as he can walk, he doesn’t need them. Otherwise, they can adversely impact his growth.’ (Mother, China, Yue et al.^36^)

The authors of the study in India ascertained that none of the mothers in their study followed the correct dosage recommendations when giving iron supplementation to their children.^33^

**Finding 2: Lack of access to reliable information on anaemia, nutrition and iron supplementation (moderate confidence):** The study in China reported that although mothers were keen to gain knowledge of nutrition and anaemia, there was a lack of awareness of iron supplementation programmes in their communities.^36^ As a result, some relied on their experiences and those of their families and friends as their source of information regarding the nutrition of their children:

‘I get lots of advice from my family, but they all say something different. I usually listen to whomever I disagree with least.’ (Mother, China, Yue et al.^36^)

Similarly, other caregivers mentioned that they rely on media for nutrition information:

‘I don’t think I have enough good information to take care of the baby. I don’t talk to other people about the baby. And I see ads about babies on TV sometimes, but I don’t understand them.’ (Grandma, China, Yue et al.^36^)

**Finding 3: Caregivers’ perceived benefits and harms of iron supplements (high confidence):** Four studies revealed that some caregivers understood the importance of iron supplementation and focussed on its benefits, which was attributed to the adequate information they had received.^33,34,36,37^ For example, some mothers expressed many benefits of iron supplementation, such as improving their children’s health:

‘… so many benefits have occurred … She became more fat,’ (Mother, India, Young et al.^34^)

Other caregivers indicated that iron supplements may improve their children’s weight and overall health, such as gaining strength, intelligence or recovering from ill health:

‘She is little more active than before. She is healthy. She has gained weight and now she is maintaining a good weight. She talks well after giving iron supplements.’ (Mother, India, Kwon et al.^32^)

This understanding of the benefits of iron supplementation appeared to increase many of these caregivers’ acceptance of it for their children.

In contrast, the four studies also revealed that many caregivers were concerned about the potential harms of iron supplementation.^33,34,35,37^ Specifically, these concerns related to the side effects and complications of iron supplementation, including stomach pains, vomiting, diarrhoea, constipation and teeth staining. These concerns contributed to reducing some caregivers’ acceptance of iron supplementation. In some instances, caregivers stopped giving iron supplementation to their children for fear of these complications. For example, one mother in India stopped giving iron supplements after her child vomited after consuming it:

‘When she is vomiting, I gave with much difficulty, and then I stopped giving.’ (Mother, India, Kwon et al.^33^)

**Finding 4: Iron supplementation palatability or tolerability (high confidence):** Many caregivers in studies from low- and high-income countries raised concerns regarding iron supplementation palatability and tolerability and suggested that certain types of iron supplementation were preferable to other types.^33,34,35,37^ Various caregivers appeared to be more accepting of iron syrups compared to other forms of supplementation, such as powders. For some, this was based on a belief that syrup was more suitable for children. Similarly, others felt that syrup would enable absorption of the full treatment dose in contrast to powders, where dose consumption was perceived to be dependent on the quantity of food consumed.^34^ For example, a mother commented that she liked the syrup as her child:

‘… was not wasting … [*and*] use to drink (the iron syrup) easily.’ (Mother, India, Young et al.^34^)

Similarly, some mothers found it challenging to give iron supplements to their children because of its unpalatable taste, as reported by an HCW in Argentina:^35^

‘… I don’t know if I’d call it intolerance because some mothers say, “[*I*]t tastes funny and my child doesn’t want to take it …”.’ (Paediatrician, Argentina, Sguassero et al.^35^)

Various HCWs suggested that a better-tasting or flavourful iron supplementation could enhance caregivers’ acceptance of it.^35^

**Finding 5: Relationships between healthcare workers and caregivers (high confidence):** Good relationships and trust between HCWs and caregivers reinforced some caregivers’ acceptance of iron supplement interventions. Studies from India and China (Kwon et al.^33^; Yue et al.^34^) reported on caregivers’ views about their relationship with HCWs,^33,36^ while a study in Argentina reported HCWs’ views about their relationship with caregivers.^35^

The study in India found that some caregivers’ adherence was influenced by their relationship with HCWs.^33^ In this study, caregivers were appreciative of HCWs’ support and effort to deliver iron supplementation. Similarly, caregivers confirmed the importance of HCWs distributing iron supplementation, highlighting how LHWs, such as village health workers (VHWs), fill iron supplementation implementation gaps and challenges, such as accessibility issues. Some mothers reported that they relied on HCWs to access iron supplements on their behalf:

‘It is not difficult for me to collect the iron. I will go to the clinic and collect it. If I cannot go, the health worker comes and gives me the supplement. According to the weight, she will give.’ (Mother, India, Kwon et al.^33^)

However, the study from China found contrasting perceptions by caregivers regarding their relationships with HCWs.^36^ In this instance, caregivers believed they received inadequate support, advice and/or provision of supplementation from HCWs, which in turn reduced their acceptability of the intervention. For example, a mother reported that the doctors were not providing the nutrition information that she needed to take care of her child:

‘We’ve taken the baby to the village clinic before when he had a cold. But he’s never gone to the doctor or to get a health checkup. Doctors here don’t give nutrition advice; they just cure small problems.’ (Grandmother, China, Yue et al.^36^)

The issue of culture and its influence on iron supplementation acceptability also emerged in the US.^37^ It was reported that cultural and language differences between clinicians and caregivers negatively affected their relationship, which subsequently negatively affected caregivers’ acceptance of iron supplementation. Specifically, a lack of understanding of each other’s backgrounds and traditional practices, as well as poor communication, was found to hinder the delivery of community-based iron supplementation interventions.

**Finding 6: Community-based education and support (moderate confidence):** Various HCWs suggested that community-based education and support could help raise awareness and address caregivers’ concerns about iron supplementation, and in turn, enhance their acceptance and uptake of iron supplementation. For example, it was proposed that HCWs-caregiver workshops could be implemented to raise awareness about iron supplementation and iron prevention more broadly, strengthen HCWs-caregiver relationships and provide caregivers with an opportunity to discuss their concerns regarding iron supplementation with HCWs:

‘Yes, I guess having workshops would help a lot to the relationship and general health problems because it brings the health care team and the community together, and it’s a more informal setting, outside the office and that alone is very helpful … that would be ideal, having workshops.’ (HCW, Argentina, Sguassero et al.^35^)

Another proposed community-based activity was mass education campaigns, focussing on iron supplementation awareness-raising but also broader anaemia and iron deficiency prevention initiatives, such as the importance of healthy diets, physical exercise and encouraging vegetable gardening. Some HCWs also suggested establishing peer support groups for caregivers whose children are on iron supplementation could be beneficial. As one HCW indicated:

‘… besides mothers may exchange ideas about their habits and experiences so that they see they are not alone, that many of their peers are going through the same or something similar, that would be ideal, having workshops.’ (HCW, Argentina, Sguassero et al.^35^)

#### Theme 2: Barriers and facilitators to the feasibility of iron supplementation in the prevention of anaemia and iron deficiency among young children

**Finding 7: Healthcare workers’ knowledge of anaemia and iron supplementation (moderate confidence):** In three studies, it was reported that HCWs understood that anaemia was a problem in the communities they worked in and were engaged in several projects to address this problem.^32,35,37^ Some drew resources from the World Health Organization (WHO) and other published literature on the topic and demonstrated adequate knowledge of diagnosis, causes and risk factors of anaemia in children. For example, a paediatrician in Argentina described his knowledge of anaemia prevalence and how it has evolved over the years:

‘Well, in the setting where I work, i.e., primary health care …, in recent years it has been associated with poor nutrition rather than undernutrition … at least in the past eight or nine years, when I was trained in pediatrics, you’d see it in association with undernutrition, you’d see malnourished children with a very severe anaemia; however, now I practically see no undernourished kids but I have a lot of anaemia cases, and it’s associated to undernutrition or you may even see obese children with anaemia.’ (Paediatrician, Argentina, Sguassero et al.^35^)

Despite not being able to cite prevalence statistics, most HCWs were aware of how common the condition was. Many believed that with the knowledge they had, they would be able to manage iron deficiencies in their settings.^35,37^

**Finding 8: Lack of adequate resources and support for healthcare workers (moderate confidence):** Lack of resources and supervision was found to hinder the performance and motivation of HCWs, particularly LHWs such as VHWs. For example, in one of the Indian studies, the authors demonstrated that performance in iron supplementation programmes was impacted by the quantity and complexity of project administration required from VHWs, such as recording their home visits.^33^ Moreover, their motivation and remuneration influenced how they participated in the programme. In addition, their performance was shaped by having time for the work and whether they had to travel to attend VHW meetings at the clinic. Monthly supervisory meetings where VHWs could report back on their work and restock their supplies were reported as being important for how they participated in the programme. Kwon et al.^33^ also described how VHWs reported how demanding some programmes were, such as being expected to attend monthly meetings, familiarity with the iron supplementation programme, travelling to the clinic and building relationships with the village mothers:

‘We only have time to collect the iron supplements once a month, we have our own work. It is harder for us to check them often.’ (VHW, India, Kwon et al.^33^)

‘The VHW from that village may not have come to this meeting. So, she will not know about this. Then she wouldn’t be able to go to her village and tell people about it.’ (VHW, India, Kwon et al.^33^).

**Finding 9: Integration of iron supplementation into routine healthcare (moderate confidence):** The studies conducted in Argentina and the US suggested that oral iron supplementation uptake could be enhanced by integrating it into everyday consultations between HCWs and caregivers.^35,37^ Specifically, it was suggested that educational talks could be offered in the waiting areas, and educational handout material be offered to caregivers during these consultations. It was also recommended that routine anaemia screening could happen during consultation. Moreover, HCWs emphasised the importance of being persistent in encouraging caregivers to adhere to iron supplementation, as it involves long-term treatment. Though not offering specific activities, some paediatricians thought it important to strengthen caregivers’ linkage with health facilities:

‘Yes, I guess having workshops would help a lot to the relationship and general health problems because it brings the health care team and the community together, and it’s a more informal setting, outside the office and that alone is very helpful … that would be ideal, having workshops …’ (Paediatrician, Argentina, Sguassero et al.^35^)

‘… besides mothers may exchange ideas about their habits and experiences so that they see they are not alone, that many of their peers are going through the same or something similar, that would be ideal, having workshops …’ (Paediatrician, Argentina, Sguassero et al.^35^)

**Finding 10: Integration of iron supplementation into lay health worker programmes and other community-based activities (moderate confidence):** Community-based healthcare providers, such as LHWs, were found to often have strong relationships with communities and in-depth knowledge of community systems. It was therefore suggested that integrating iron supplementation programmes into existing community services may improve its accessibility and in turn uptake. Authors of one of the studies in India^33^ illustrated that employing VHWs to provide iron supplementation in communities improved iron accessibility for caregivers while strengthening health services. In this study, iron supplementation was only available through KC Patty Primary Health Center (KCPPHC, a non-governmental organisation [NGO] employing VHWs), and no government programme was functioning in this setting. According to Kwon et al.,^33^ although the approach was impactful, such interventions require resources to ensure feasibility (see ‘Finding 8’; moderate confidence):

‘While the geographical barriers limited mothers’ access to the supplements, in the few more distant villages where the VHWs were very active, reported adherence was also greater, suggesting that distance barriers are modifiable by increasing the support for community-level VHWs.’ (Participant group, India, Kwon et al.^33^)

It was also suggested that iron supplementation programmes should link up and integrate with existing community structures and NGOs to form part of broader anaemia prevention campaigns, including, for example, sanitation, personal hygiene, clean water, good waste management systems, and growing vegetables and fruits. Specific examples include water, sanitation and hygiene (WASH) projects; community and household gardening; supplementary food provision to refugee populations and the Ghana School Feeding Programme (GSFP).

#### Theme 3: Equity factors shaping iron supplementation delivery and uptake

**Finding 11: Socio-economic challenges influencing supply and access (low confidence):** Healthcare workers and caregivers raised concerns about the inaccessibility of iron supplementation, including that it was not free of charge and often difficult to access because of the distances caregivers were required to travel.^32,33,35^ Authors of the studies in Ghana and India reported that caregivers who lived far from health facilities where iron supplements were stored were less motivated to use them as compared to those living close by.^32^ Specifically, these studies found that a lack of transport fare, poor access to transport and lack of time to travel were major barriers to regular iron supplementation use among caregivers. Those who lived far from facilities and in remote areas had reduced access to iron supplements. In addition, some caregivers did not have enough money to purchase iron supplements regularly, which in turn resulted in inconsistent usage.^33^ Furthermore, an LHW described the challenges they faced while working with poor caregivers:

‘What I think is that if they [*caregivers*] don’t have money, we can pay for them instantly, and [*t*]hen provide everything for them.’ (VHW, India, Kwon et al.^33^).

## Discussion

### Characteristics of the evidence base

To the best of our knowledge, this is the first review to synthesise qualitative evidence on factors that influence the uptake and provision of iron supplementation as a public health intervention in young children for the prevention of iron deficiency and IDA.

Evidence to inform the review was sparse, with only six studies included. This suggests a gap in qualitative research on factors influencing the uptake and provision of preventive oral iron supplementation in young children. Only two studies included in this review focussed exclusively on preventive supplementation, both being conducted in India, where preventive iron and folic acid (IFA) supplementation is widely implemented for young children.^33,34^

The included studies explored the perceptions of caregivers and other stakeholders, such as programme officers and community stakeholders. Most of the studies explored the perceptions of caregivers, but all explored the perceptions of HCWs. These results were anticipated because mothers and grandparents are often primary caregivers of the children in this age group and are primarily responsible for their children’s health.^38^ In addition, HCWs are currently the only cadre to deliver iron supplements as recommended by WHO.^13^

### Key qualitative findings

#### Factors that influence caregivers’ acceptability of oral iron supplementation

Our findings indicate that caregivers’ knowledge of iron supplementation, their relationships with HCWs and their perceptions of the benefits and harms, as well as the palatability and tolerability of iron supplements, can act as barriers or facilitators to caregiver acceptance (4 findings; high confidence). These findings are similar to an existing study on a vitamin A supplementation programme that also found that caregivers have limited knowledge of supplements that can influence uptake.^39^ In addition, other studies have also found that good relationships and trust between HCWs and caregivers may contribute to increasing caregivers’ acceptance of, or adherence to, iron supplementation, while poor relationships between HCWs and caregivers may contribute to reducing some caregivers’ acceptance of, or adherence to, iron supplementation.^40^ Previous studies have also reported side effects of oral iron-containing supplements for the prevention and management of anaemia, such as constipation, diarrhoea, stomach cramps and vomiting.^41,42^ Our findings around increased acceptance of caregivers when the intervention was administered as a syrup and not a powder are consistent with a study reporting that powders are found to be unpalatable, and caregivers generally prefer syrups as they are easy to measure and swallow by children.^19^

We found some caregivers were concerned by a lack of access to reliable information on anaemia, nutrition and iron supplementation, which affected their acceptance; however, HCWs were found to consider community-based education and support to play an important role in caregiver acceptance and uptake of iron supplementation (2 findings; moderate confidence). Our review found that many caregivers were unaware of community programmes – often relying on their own experiences, or those of their families and friends, as well as media sources of information. In contrast, a study on vitamin A supplementation reported HCWs and trained community health volunteers as the main sources of nutrition information.^43^ Our findings did, however, align with literature showing that areas where caregivers received support and were more aware of micronutrients had better coverage and uptake of such interventions.^19,44^

#### Factors that influence the feasibility of implementing oral iron supplementation programmes

We found that HCWs with adequate knowledge about anaemia and its management, as well as the integration of iron supplementation into routine healthcare, LHW programmes or community-based activities, enhance the feasibility of implementing iron supplementation programmes; however, a lack of resources, support and supervision acts as a barrier to the effective delivery of iron supplementation (4 findings; moderate confidence). While existing literature indicates incorporating iron supplementation into routine micronutrient and deworming delivery programmes for infants improves coverage, the efficacy of these approaches may be hampered by a lack of financial and other resources, particularly in under-resourced settings.^45,46^

#### Equity issues shaping oral iron supplementation delivery and uptake

Our findings, though informed by sparse data, suggest that caregivers living in resource-limited settings faced several socio-economic challenges in accessing iron supplements, and as such, contributed to irregular or limited usage of iron supplementation among many of these caregivers (1 finding; low confidence). Similarly, previous studies found economic status and distance from health facilities impact the uptake of micronutrient supplementation interventions. Generally, the poor adherence and uptake of supplementation in children from poor households and those far from health facilities^39^ suggests a need to make services more accessible to these vulnerable groups.

### Strengths and limitations of the review

Strengths of this review include its global scope, comprehensive search strategy targeting key databases, as well as methodological rigour in identifying studies, synthesising data and assessing the confidence in the review findings. However, because of time constraints, we only included studies that were published in English, thereby potentially missing important findings of studies published in other languages.

### Implications for research

While our review did identify important factors affecting the implementation of iron supplementation, only six studies were eligible, as they included perspectives around preventive oral iron supplementation in young children. Furthermore, data relating to equity were sparse, and no data were from SA specifically. Therefore, more qualitative research on the perspectives of stakeholders around factors influencing the delivery and uptake of iron supplementation when it is being used as a public health intervention to reduce the prevalence of anaemia and/or iron deficiency is needed. This would enable more definitive conclusions regarding the acceptability, feasibility and equity considerations of this intervention. Most of the studies were assessed as moderate quality with the adapted version of the CASP tool, but there were some concerns related to the methodological limitations of the evidence base. All included studies were based on in-depth interviews and focussed group discussions.

It should be noted, however, that the confidence in most of our findings was affected by concerns related to methodological limitations of the primary studies, incoherence because of ambiguous data and concerns about data adequacy. Further high-quality primary studies collecting qualitative data may add additional depth to the data, thereby increasing the confidence in our findings while also potentially changing the findings themselves. Lastly, future qualitative studies need to improve on reflexivity and sampling strategy.

### Implications for practice

Based on our findings of this review, a full set of factors which policymakers may consider when developing and implementing the intervention are further described in Table 4. Firstly, it could be beneficial for policymakers to take into consideration caregivers’ preferences and the characteristics of the supplements (e.g. determining preferences for powder or syrup and managing concerns around potential side effects). Furthermore, it may be important to consider what resources HCWs would require to effectively implement an iron supplementation programme (e.g. standard operating procedure, transportation and adequate stock). Training needs should also be considered to ensure HCWs have the essential skills and knowledge for effective delivery.

**TABLE 4:** Factors to consider for the planning, implementation or management of a prophylactic oral iron supplementation programme or intervention.

Item numbers	Questions
1	When designing the iron supplements, have you thought about the caregiver’s preferences and the characteristics of the supplements? For instance, would caregivers prefer iron supplements in the form of powder or syrup? Consider how to manage caregivers’ concerns regarding potential side effects and complications of iron supplementation
2	What are the resources that healthcare providers would need to effectively implement and deliver iron supplements? For instance, what are the standard operating procedures, transportation and the supplements required?
3	When selecting HCWs for iron supplements, particularly LHWs, have you considered how they will be perceived by members of the community in which they will work, for instance in terms of their sociocultural or economic background or gender? For example, could their social or economic status or gender deter some members of the community from accessing iron supplementation programmes?
4	Have you considered whether collaboration with community-based organisations could increase the credibility and acceptance of iron supplementation? Remember that the success of this involvement is only likely to be useful where community-based organisations are actively involved
5	Have you provided LHWs with sufficient means of transportation, where necessary?
6	Have you provided HCWs with the essential skills and knowledge for them to effectively deliver iron supplements?
7	Have you considered whether it might be possible for iron supplementation to be integrated into routine healthcare services?
8	Have you considered whether community members, particularly caregivers, are aware of the range of activities being performed by LHW programmes in iron supplementation programmes? For example, are they aware that LHWs can deliver the supplements from health facilities to households?
9	Have you considered how to ensure good working relationships between professional health workers and LHWs? For instance, are other professional healthcare providers encouraged to be respectful, supportive and responsive towards LHWs?
10	Has the use of LHWs in iron supplementation delivery added to the workload of other healthcare providers, for instance, because of additional tasks such as evaluation and supervision? Or do they perceive LHWs as lessening their workloads and bringing complementary skills, knowledge and experience?
11	Have you thought about effective approaches to raise awareness and/or educate the communities, particularly caregivers, about anaemia and iron supplementation?

HCWs, healthcare workers; LHW, lay health worker.

Healthcare workers should also be empowered to establish strong relationships with caregivers to facilitate intervention acceptance and adherence. How HCWs are perceived (e.g. sociocultural background and gender) could play an important role in whether the community accesses iron supplementation programmes. Iron supplementation programmes could be integrated into routine healthcare services or other LHW programmes. Ensuring good working relationships with cadres may be critical for successful programme implementation.

Finally, the establishment of strong collaborations with community-based organisations and community-based education activities, such as mass education campaigns or peer support groups, may help to raise awareness and increase the credibility and acceptance of iron supplementation.

## Conclusion

Our findings highlighted several factors relevant to acceptability and feasibility that may influence the uptake and provision of preventive oral iron supplementation as a public health intervention in young children. Our review found that oral iron supplementation is mostly acceptable to caregivers and feasible to provide if HCWs have the necessary resources and support. Though socioeconomic challenges around access were identified, there was a paucity of data related to equity.
